# Supplementary material for: NS1 Protein N-Linked Glycosylation Site Affects the Virulence and Pathogenesis of Dengue Virus
Source: Vaccines (Basel). 2023 May 8;11(5):959. doi: 10.3390/vaccines11050959 (PMC10221952; doi:10.3390/vaccines11050959)
Supplement: Supplementary file 1 [file vaccines-11-00959-s001.zip › vaccines-2327179-supplementary-Table S1&2.pdf]

Supplementry materials

Table S1. Cloning primers and digestion sites for dengue virus NS1 protein with mutations at the N-glycosylation site.

Mutation site	Primer	Template	Primer sequence (5’→3’)	Restriction sites of the vector
N130A	F1	Ban18HK20	GCTACAATTAATACATAACCTTATGTATCATACACATACG	AscI/AflII
	N130A-R1		TATTAGAAATGTGCTAgcTTTTGCTTCTGGAGTAAAGATC	
	N130A-F2	Ban18HK20	TTTACTCCAGAAGCAAAAgcTAGCACATTTCTAATAGACG	
	R2		CATCCCATACCAGCATCCATCCTCTCCCAAAAACCTTAAG	
N207A	F1	Ban18HK20	GCTACAATTAATACATAACCTTATGTATCATACACATACG	AscI/AflII
	N207A-R1		TATCTGCCAGGTCTGGgcTTTTGAGCTCTCTATCCAATAG	
	N207A-F2	Ban18HK20	ATAGAGAGCTCAAAAgcCCAGACCTGGCAGATAGAGAAAG	
	R2		CATCCCATACCAGCATCCATCCTCTCCCAAAAACCTTAAG	
N130Q	F1	Ban18HK20	GCTACAATTAATACATAACCTTATGTATCATACACATACG	AscI/AflII
	N130Q-R1		TATTAGAAATGTGCTctgTTTTGCTTCTGGAGTAAAGATC	
	N130Q-F2	Ban18HK20	ACTCCAGAAGCAAAAcagAGCACATTTCTAATAGACGGAC	
	R2		CATCCCATACCAGCATCCATCCTCTCCCAAAAACCTTAAG	
N207Q	F1	Ban18HK20	GCTACAATTAATACATAACCTTATGTATCATACACATACG	AscI/AflII
	N207Q-R1		ATCTGCCAGGTCTGctgTTTTGAGCTCTCTATCCAATAGC	
	N207Q-F2	Ban18HK20	TAGAGAGCTCAAAAcagCAGACCTGGCAGATAGAGAAAGC	
	R2		CATCCCATACCAGCATCCATCCTCTCCCAAAAACCTTAAG	
130-132QQA	F1	Ban18HK20	GCTACAATTAATACATAACCTTATGTATCATACACATACG	AscI/AflII
	130QQA-R1		TATTAGAAAcgcctgctgTTTTGCTTCTGGAGTAAAGATC	
	130QQA-F2	Ban18HK20	AGAAGCAAAAcagcaggcgTTTCTAATAGACGGACCAGAC	
	R2		CATCCCATACCAGCATCCATCCTCTCCCAAAAACCTTAAG	
207-209QQA	F1	Ban18HK20	GCTACAATTAATACATAACCTTATGTATCATACACATACG	AscI/AflII
	207QQA-R1		TATCTGCCAcgcctgctgTTTTGAGCTCTCTATCCAATAG	
	207QQA-F2	Ban18HK20	AGAGCTCAAAAcagcaggcgTGGCAGATAGAGAAAGCATC	
	R2		CATCCCATACCAGCATCCATCCTCTCCCAAAAACCTTAAG	
N130-del	F1	Ban18HK20	GCTACAATTAATACATAACCTTATGTATCATACACATACG	AscI/AflII
	N130-del-R1		GTCTATTAGAAATGTGCTTTTTGCTTCTGGAGTAAAGATC	
	N130-del-F2	Ban18HK20	TTTACTCCAGAAGCAAAAAGCACATTTCTAATAGACGGAC	
	R2		CATCCCATACCAGCATCCATCCTCTCCCAAAAACCTTAAG	
N207-del	F1	Ban18HK20	GCTACAATTAATACATAACCTTATGTATCATACACATACG	AscI/AflII
	N207-del-R1		CTATCTGCCAGGTCTGTTTTGAGCTCTCTATCCAATAGCC	
	N207-del-F2	Ban18HK20	GGATAGAGAGCTCAAAACAGACCTGGCAGATAGAGAAAGC	
	R2		CATCCCATACCAGCATCCATCCTCTCCCAAAAACCTTAAG	
N130A+N207A	Com-F1	N130A	AAGTGGCTCTGTTAAGAACC	Eco32I
	Com-R1		GCTTTCTGGTCTTTGATTGC	

	Com-F2	N207A	CCAATGAACGAAGAGCATGG	
	Com-R2		TTGTCTCCGAGCCTCCATCC	
130-132QQA+207-209QQA	Com-F1	130-132QQA	AAGTGGCTCTGTTAAGAACC	Eco32I
	Com-R1		GCTTTCTGGTCTTTGATTGC	
	Com-F2	207-209QQA	CCAATGAACGAAGAGCATGG	
	Com-R2		TTGTCTCCGAGCCTCCATCC	
N130-del+207-209QQA	Com-F1	N130-del	AAGTGGCTCTGTTAAGAACC	Eco32I
	Com-R1		GCTTTCTGGTCTTTGATTGC	
	Com-F2	207-209QQA	CCAATGAACGAAGAGCATGG	
	Com-R2		TTGTCTCCGAGCCTCCATCC	

Note: Briefly, two pairs of primers are used to amplify two fragments containing homologous sequences (e.g. F1 and N130A-R1 to amplify fragment 1, and N130A-F2 and R2 to amplify fragment 2) for subsequent homologous recombination cloning with vector fragments.

Table S2. Sequencing primers for dengue virus NS1 protein with mutations at the N-glycosylation site.

Mutant viruses	Primer	Primer sequence (5’→3’)
#1 N130A;<br>#2 N207A;<br>#3 N130Q;<br>#4 N207Q;<br>#5 130-132QQA;<br>#6 207-209QQA;<br>#7 N130-del;	E-NS1-seqF	tagggtctgcttcagtaagc
	E-NS1-seq1	cagagggaatcaacaaatgc
	E-NS1-seq2	ccttatctcaaagaggaacaag
	E-NS1-seq3	atgtgctcaggaaagttctc
	E-NS1-seq4	acacttggacagaacagtac
	E-NS1-seq5	acatgtctgtggcccaagac
	E-NS1-seqR	ccctgtccggccgatacctg
#9 N130A+N207A;<br>#10 130-132QQA+207-209QQA;<br>#11 N130del+207-209QQA.	Com-F1	AAGTGGCTCTGTTAAGAACC
	Com-R2	TTGTCTCCGAGCCTCCATCC
	Com-seq1	ATCCTAGCACTGTCACATCC
	Com-seq2	TCTCGAATTCGTGCCAATCC
	Com-R1	GCTTTCTGGTCTTTGATTGC
	Com-F2	CCAATGAACGAAGAGCATGG
	Com-seq3	CTATGGGGCTGTTATGCCTG
	Com-seq4	ATGGCTGTGGGTTTGGTCAG

Note: These are the walking sequencing primers for the amplification products of the E and NS1 regions of the dengue virus genome.
