# Supplementary figures and images for: NS1 Protein N-Linked Glycosylation Site Affects the Virulence and Pathogenesis of Dengue Virus
Source: Vaccines (Basel). 2023 May 8;11(5):959. doi: 10.3390/vaccines11050959 (PMC10221952; doi:10.3390/vaccines11050959)

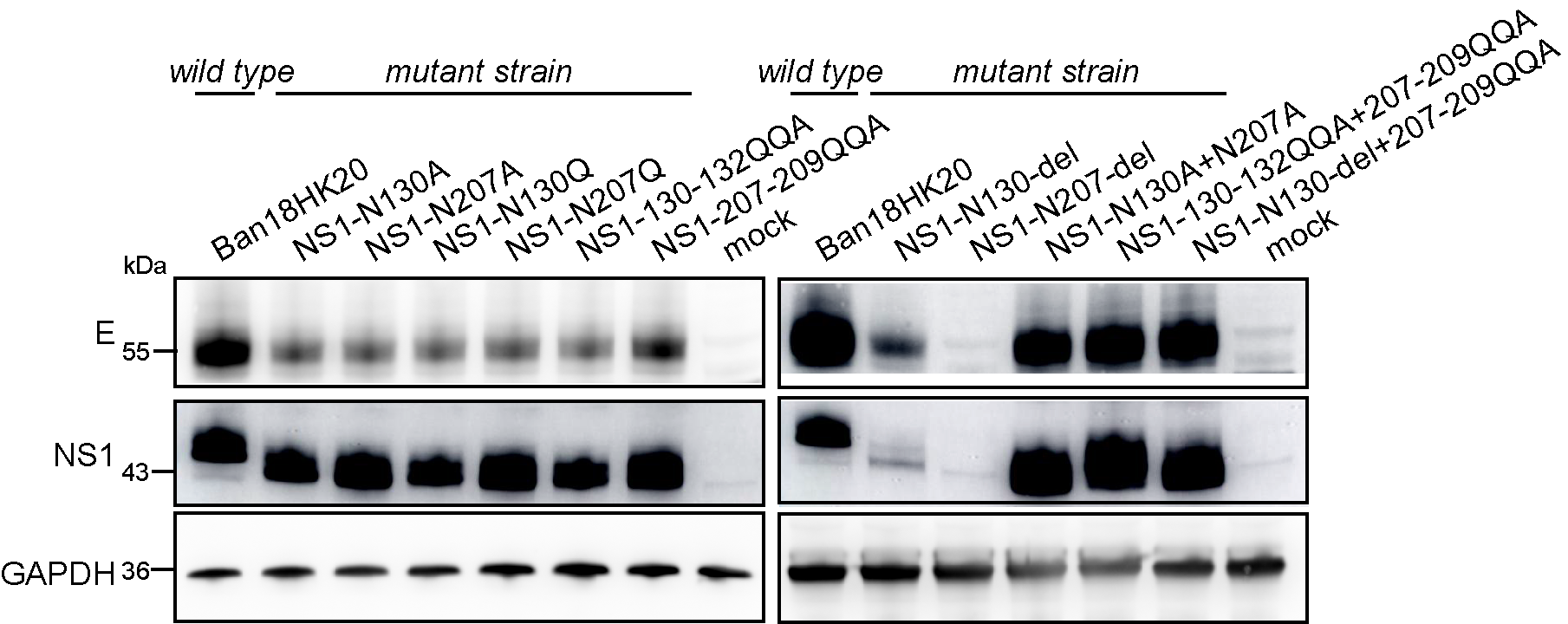

Supplement: Supplementary file 1 [file vaccines-11-00959-s001.zip › file S1-western blot/WB_intensity ratio/NS1-WB.tif]

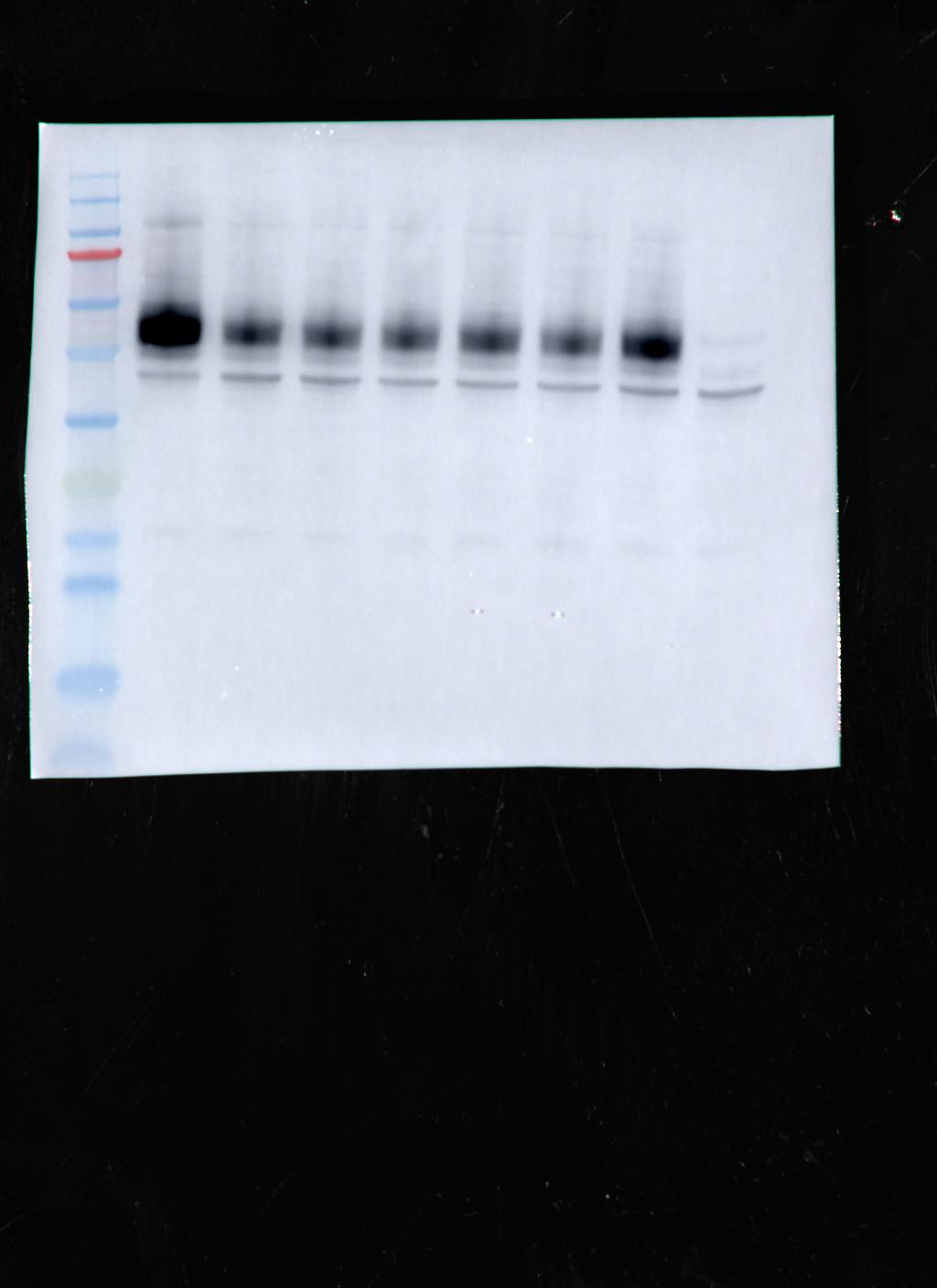

Supplement: Supplementary file 1 [file vaccines-11-00959-s001.zip › file S1-western blot/WB_whole blot/E/E(4G2)-1/20210603-4G2-1 2021.06.03_13.31.16_Ch+Marker.jpg]

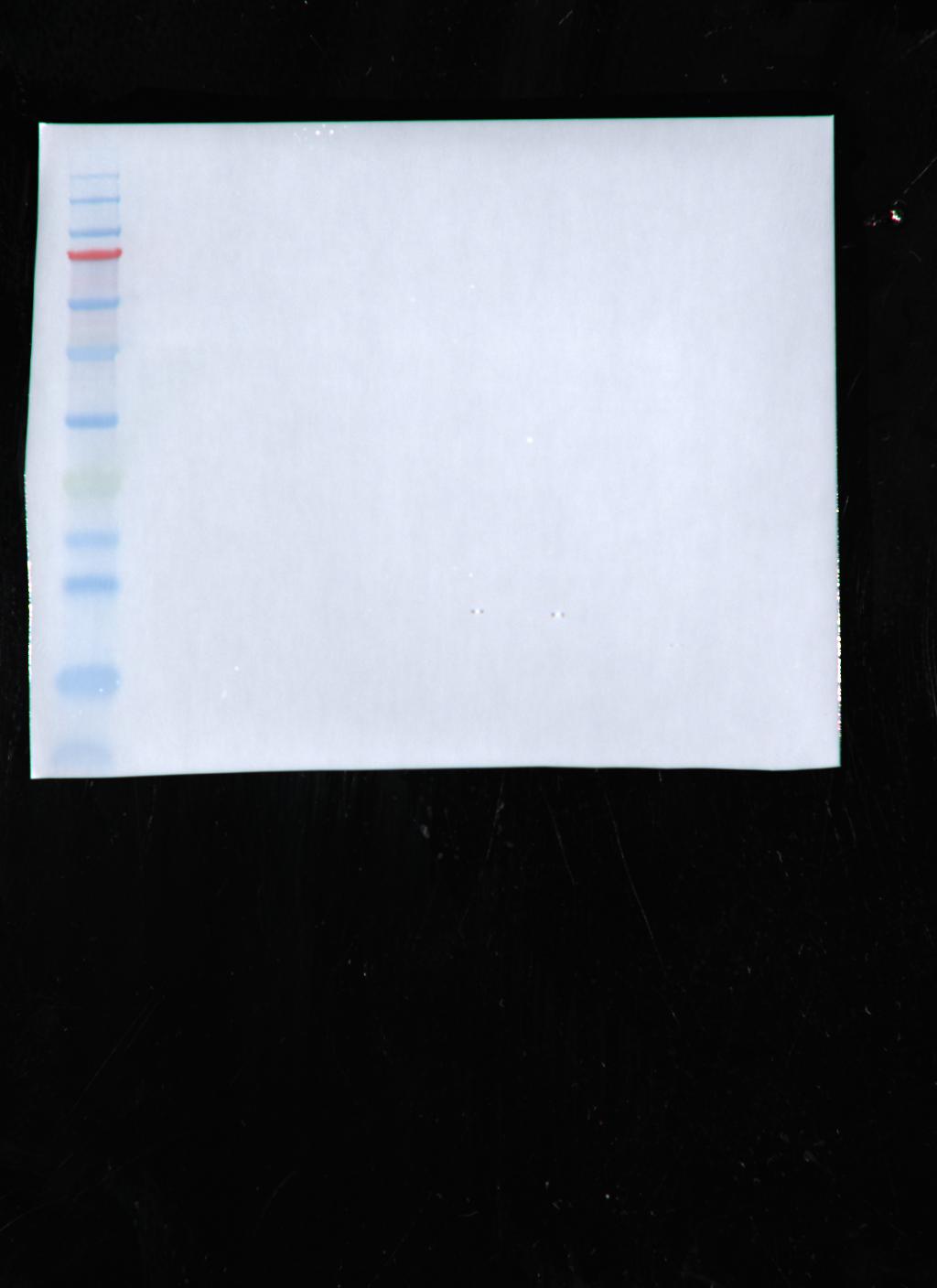

Supplement: Supplementary file 1 [file vaccines-11-00959-s001.zip › file S1-western blot/WB_whole blot/E/E(4G2)-1/20210603-4G2-1 2021.06.03_13.31.16_Ch-Marker.jpg]

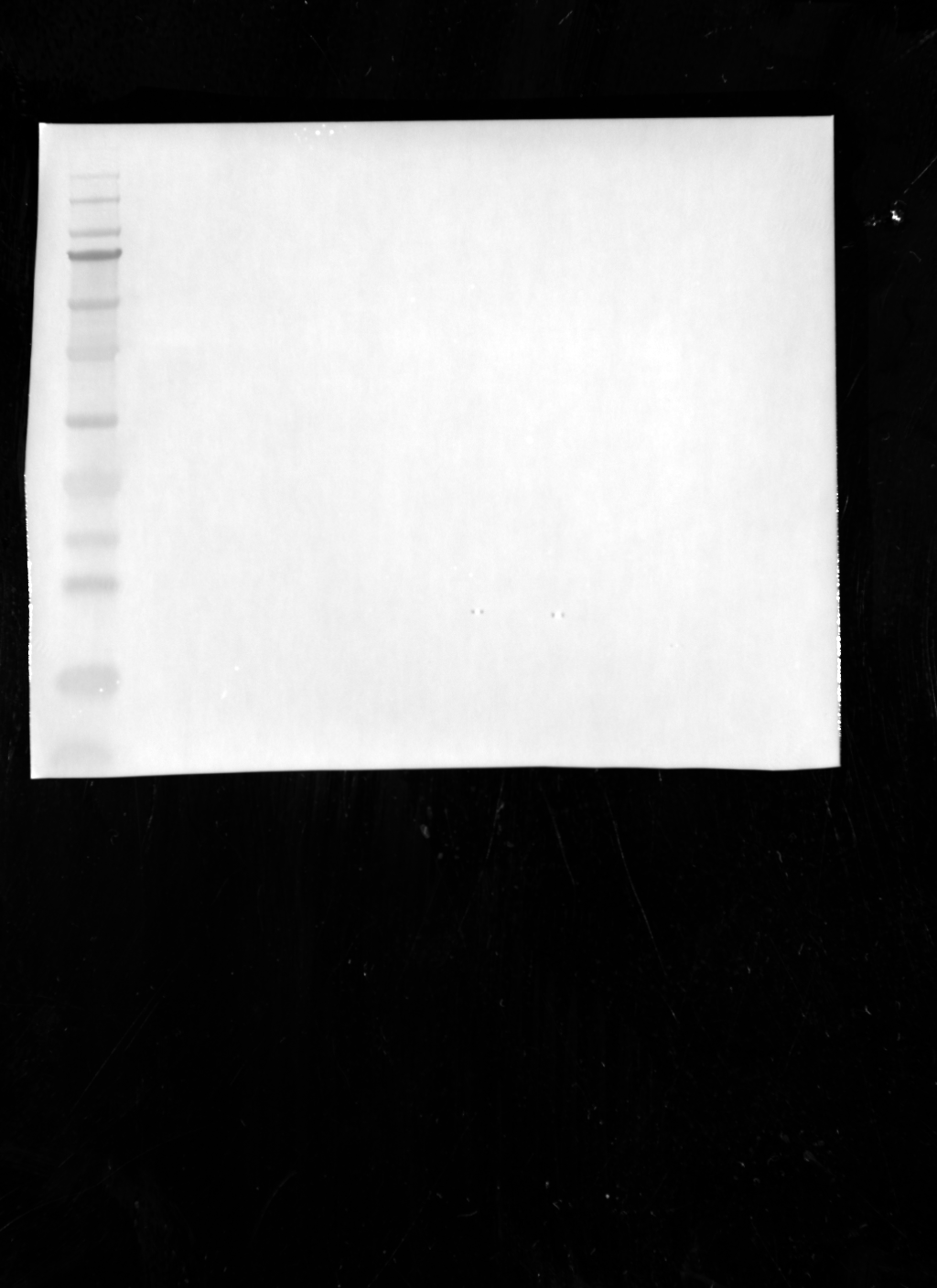

Supplement: Supplementary file 1 [file vaccines-11-00959-s001.zip › file S1-western blot/WB_whole blot/E/E(4G2)-1/20210603-4G2-1 2021.06.03_13.31.16_Ch-Marker.tif]

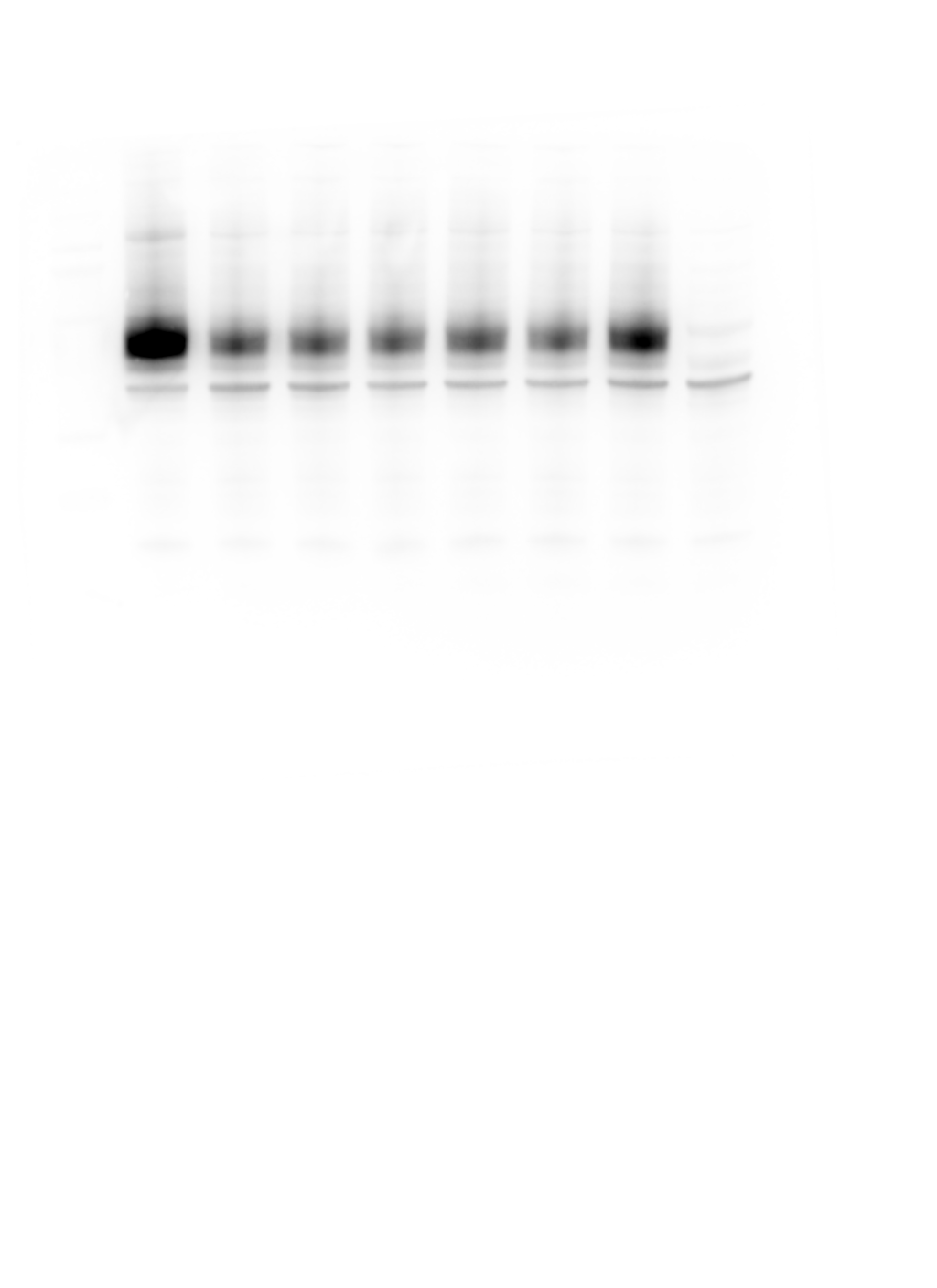

Supplement: Supplementary file 1 [file vaccines-11-00959-s001.zip › file S1-western blot/WB_whole blot/E/E(4G2)-1/20210603-4G2-1 2021.06.03_13.31.16_Ch.tif]

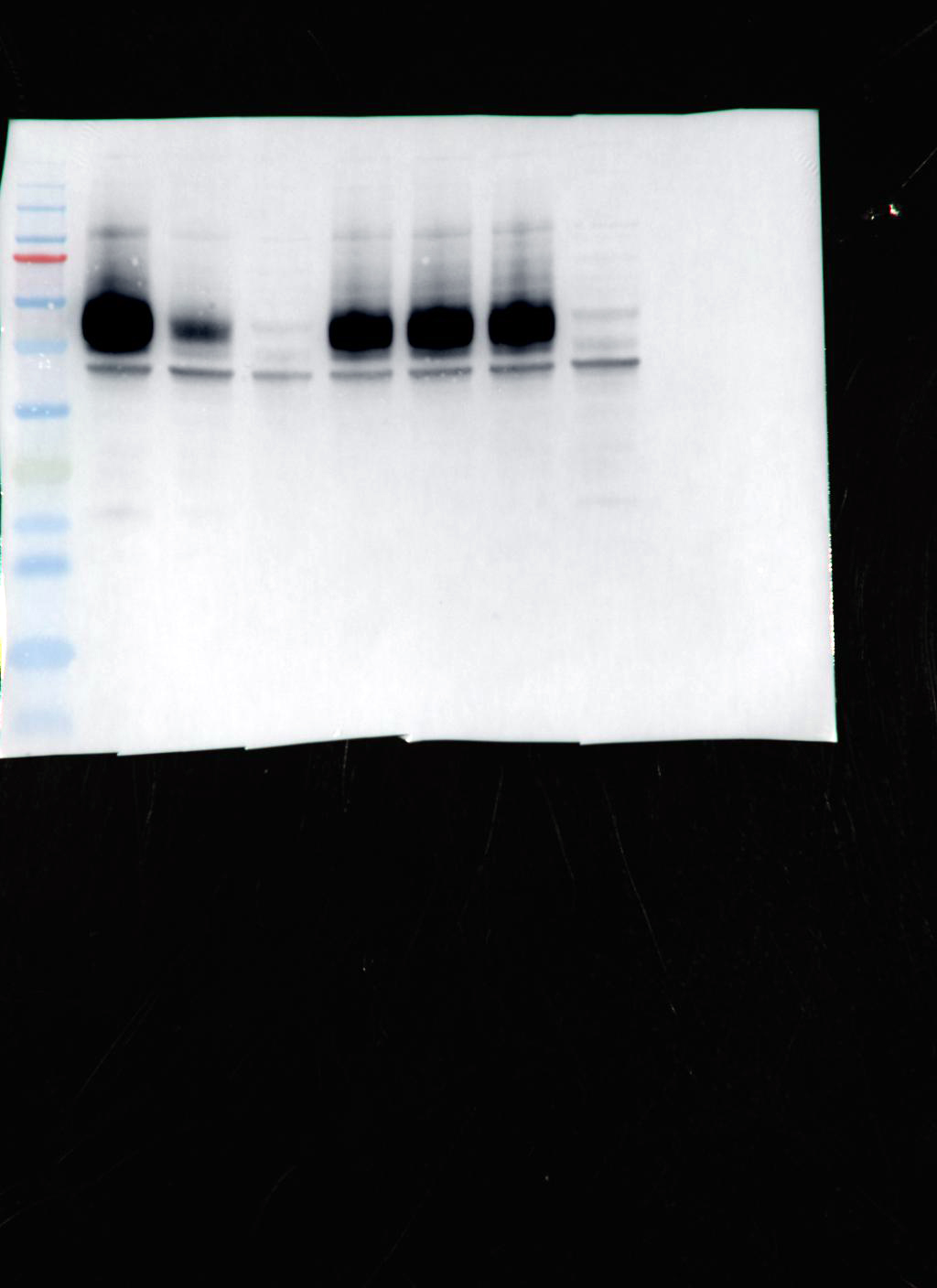

Supplement: Supplementary file 1 [file vaccines-11-00959-s001.zip › file S1-western blot/WB_whole blot/E/E(4G2)-2/20210603-4G2-2-2 2021.06.03_13.43.09_Ch+Marker.jpg]

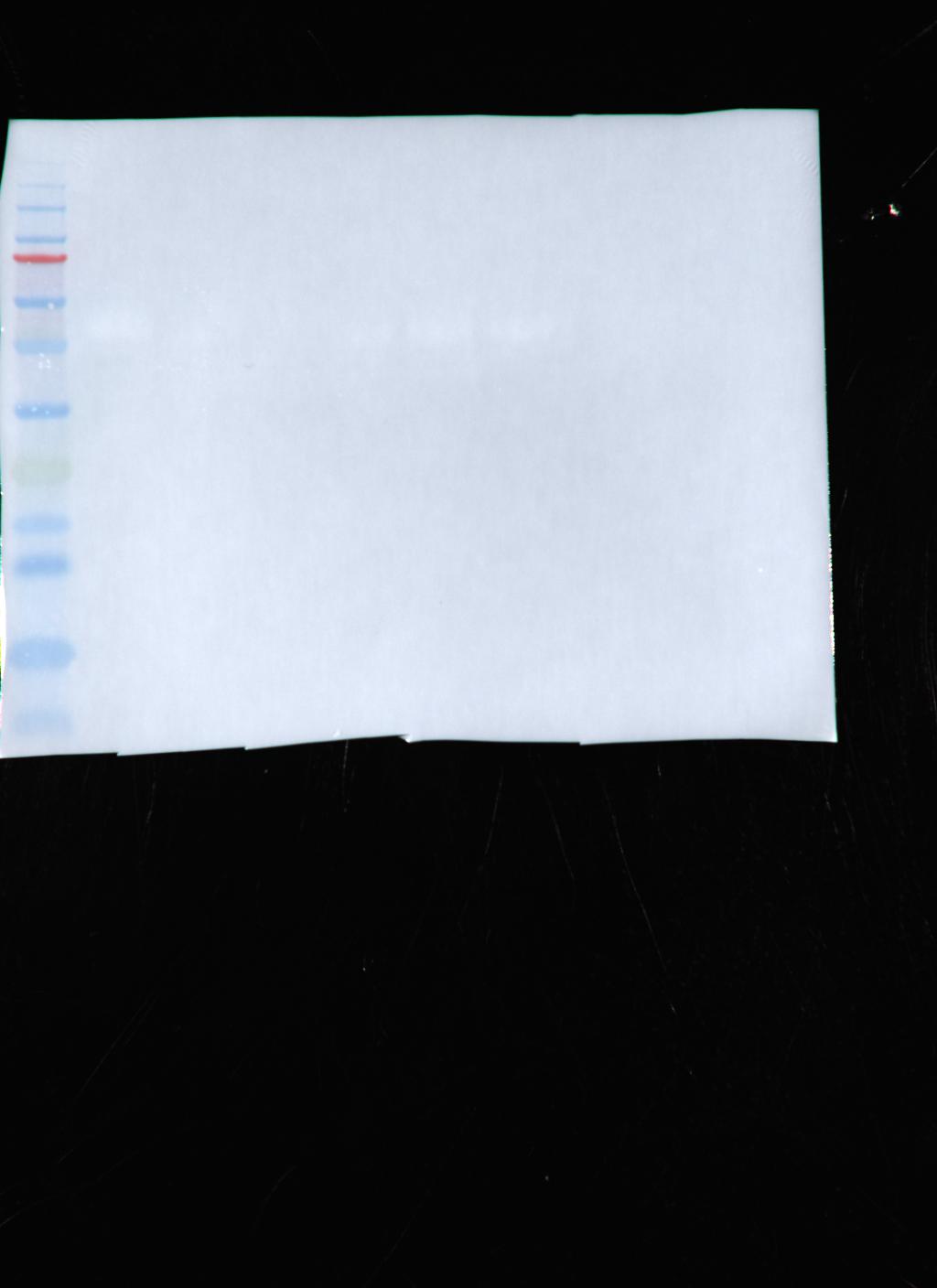

Supplement: Supplementary file 1 [file vaccines-11-00959-s001.zip › file S1-western blot/WB_whole blot/E/E(4G2)-2/20210603-4G2-2-2 2021.06.03_13.43.09_Ch-Marker.jpg]

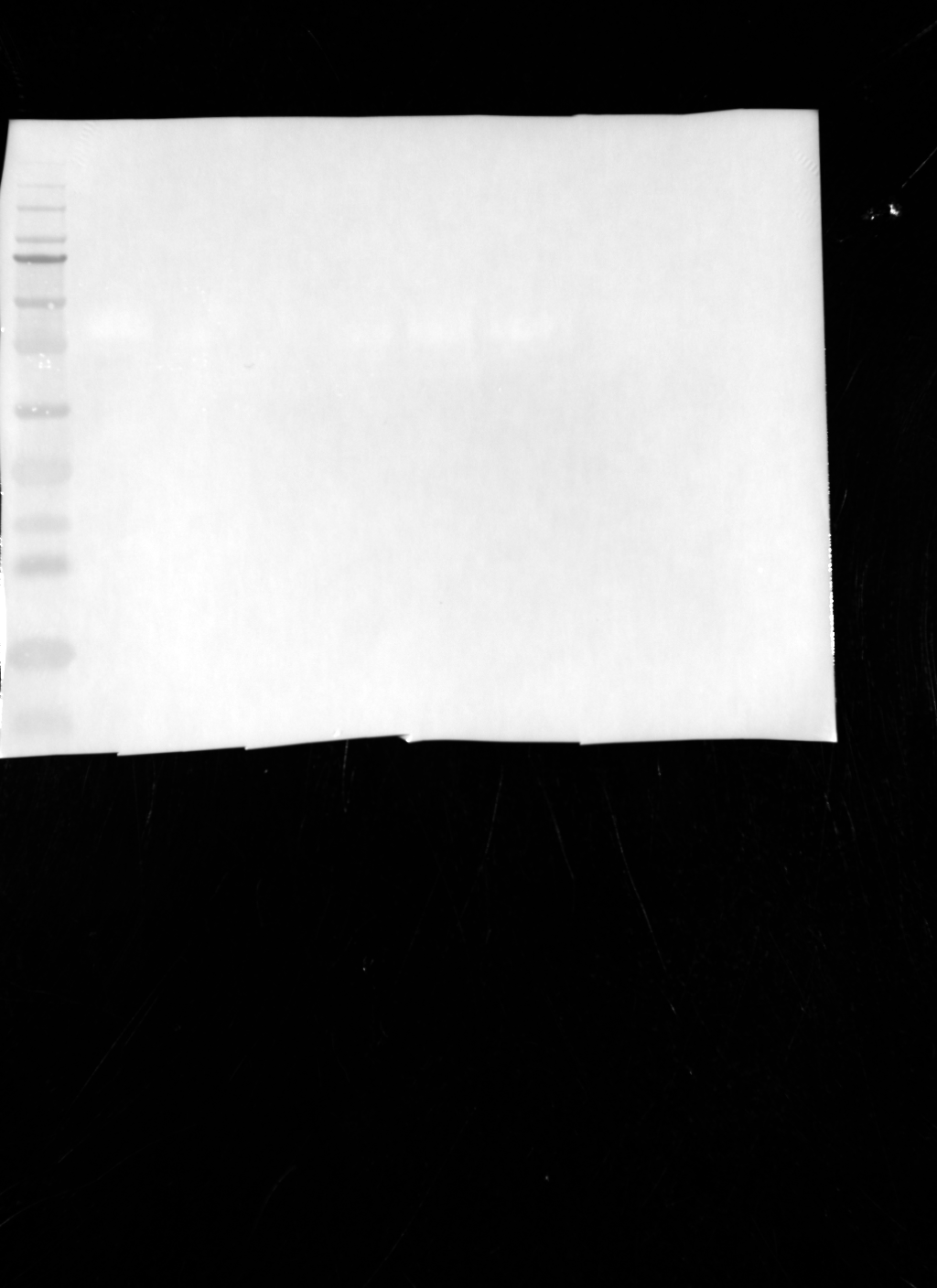

Supplement: Supplementary file 1 [file vaccines-11-00959-s001.zip › file S1-western blot/WB_whole blot/E/E(4G2)-2/20210603-4G2-2-2 2021.06.03_13.43.09_Ch-Marker.tif]

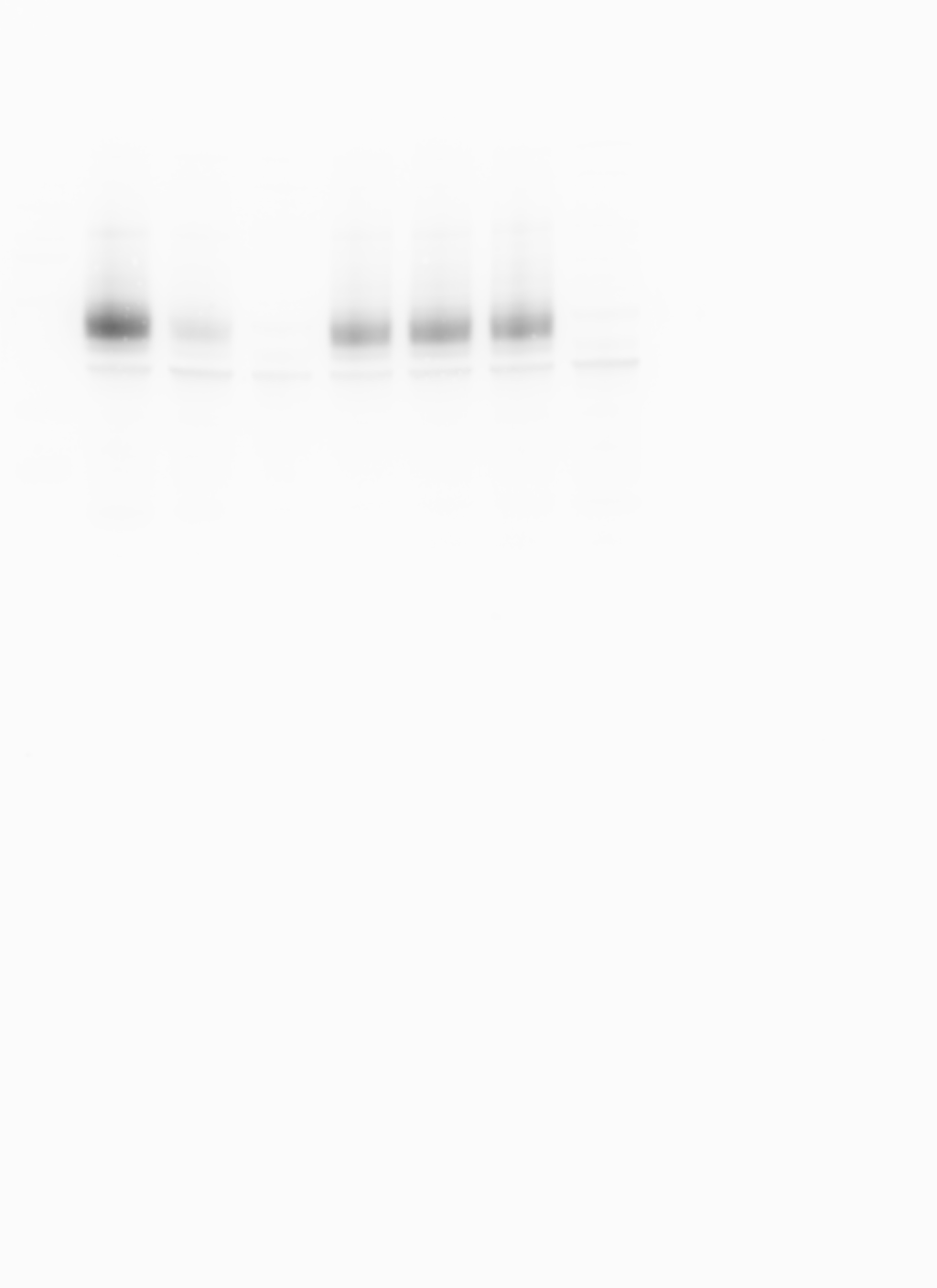

Supplement: Supplementary file 1 [file vaccines-11-00959-s001.zip › file S1-western blot/WB_whole blot/E/E(4G2)-2/20210603-4G2-2-2 2021.06.03_13.43.09_Ch.tif]

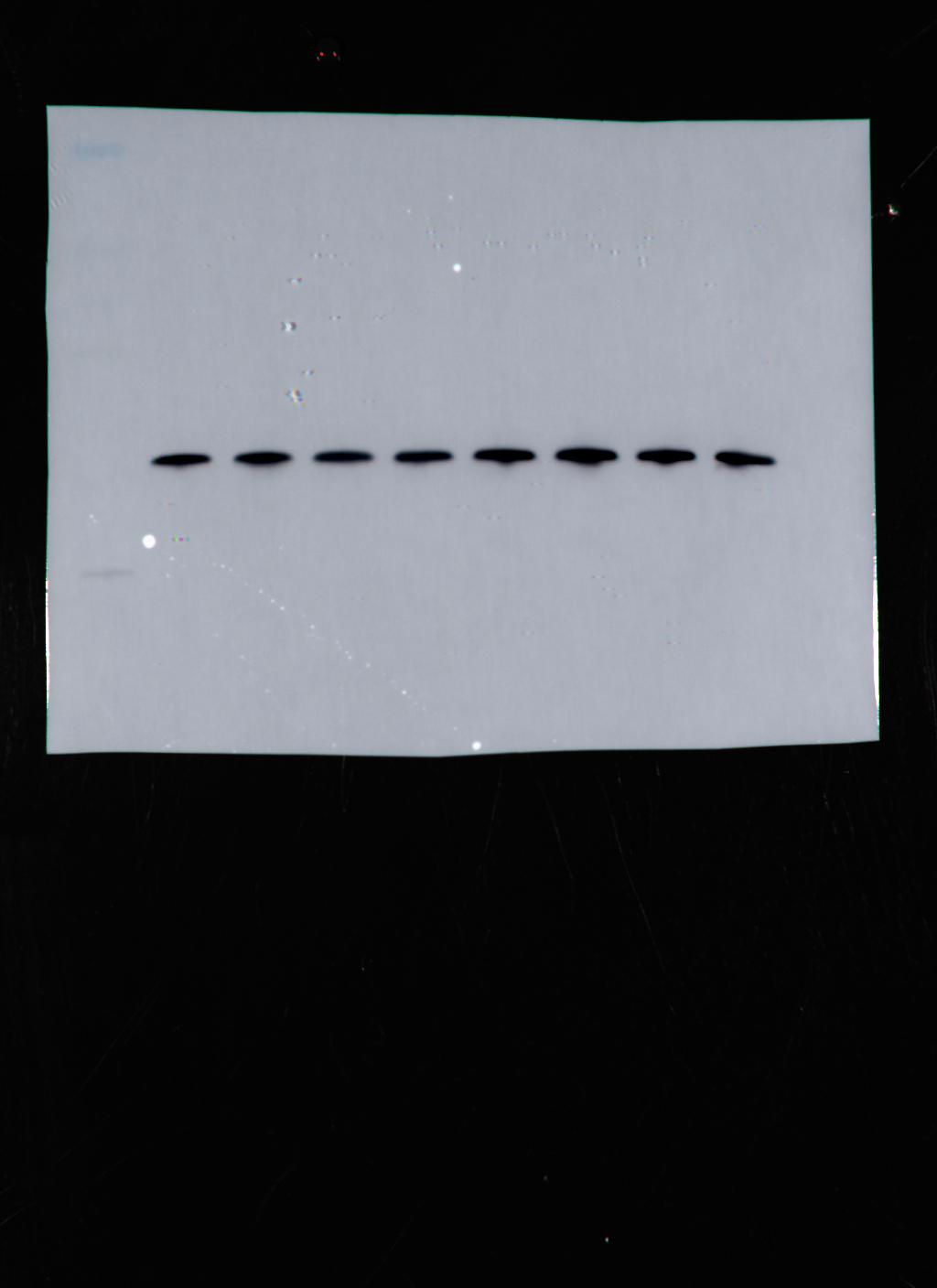

Supplement: Supplementary file 1 [file vaccines-11-00959-s001.zip › file S1-western blot/WB_whole blot/GAPDH/GAPDH-1/20210603-GAPDH-1 2021.06.03_14.07.35_Ch+Marker.jpg]

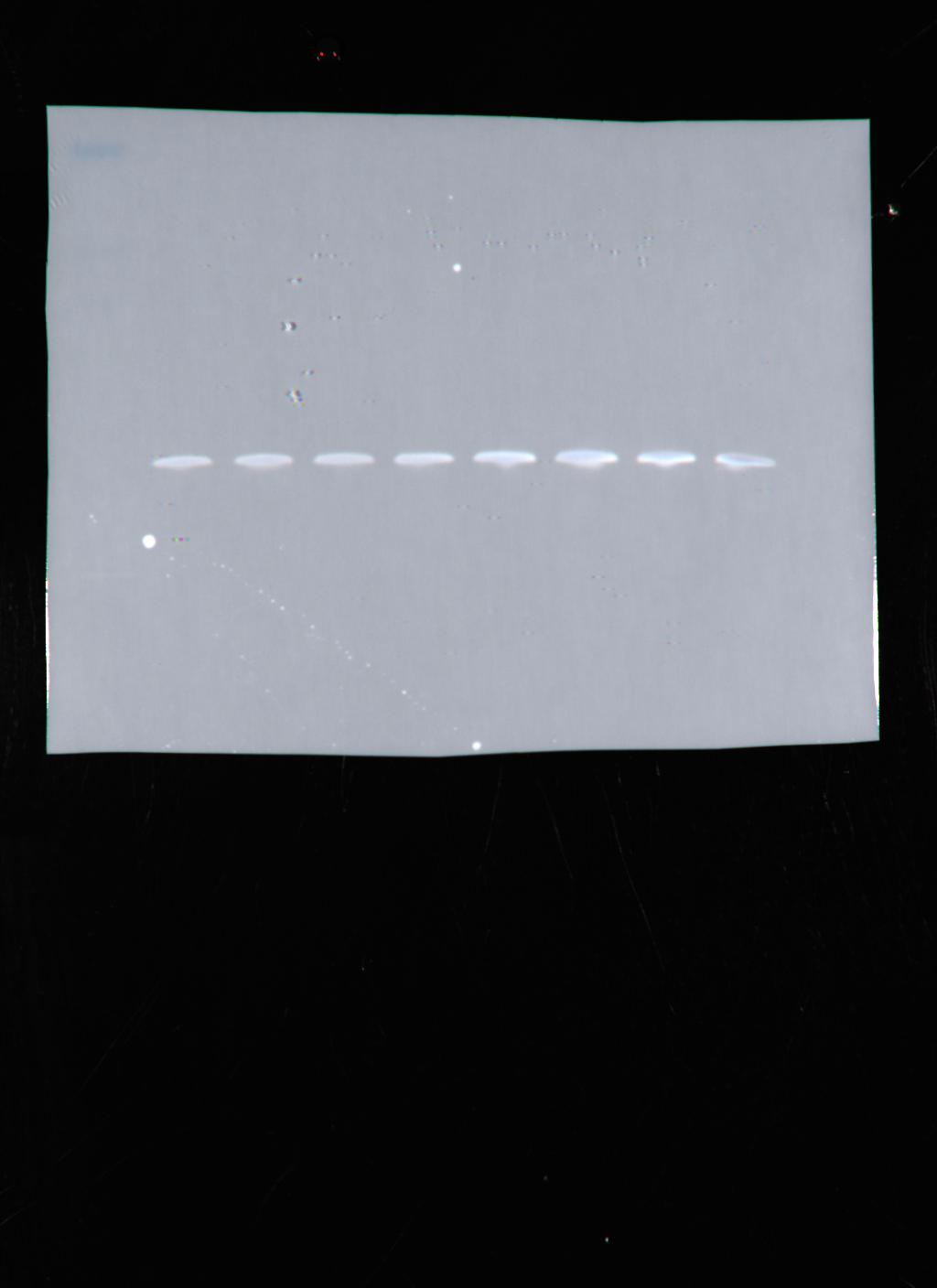

Supplement: Supplementary file 1 [file vaccines-11-00959-s001.zip › file S1-western blot/WB_whole blot/GAPDH/GAPDH-1/20210603-GAPDH-1 2021.06.03_14.07.35_Ch-Marker.jpg]

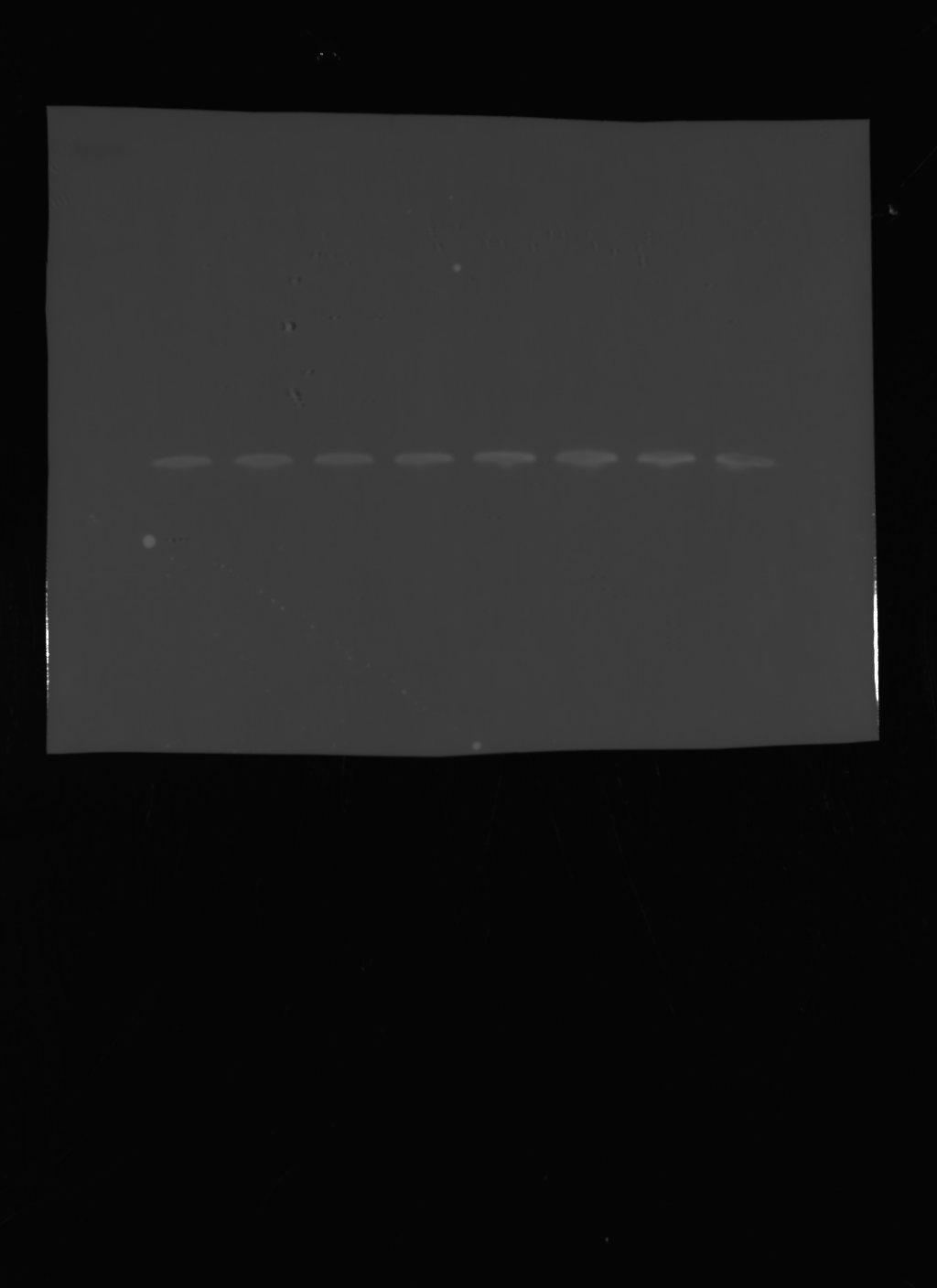

Supplement: Supplementary file 1 [file vaccines-11-00959-s001.zip › file S1-western blot/WB_whole blot/GAPDH/GAPDH-1/20210603-GAPDH-1 2021.06.03_14.07.35_Ch-Marker.tif]

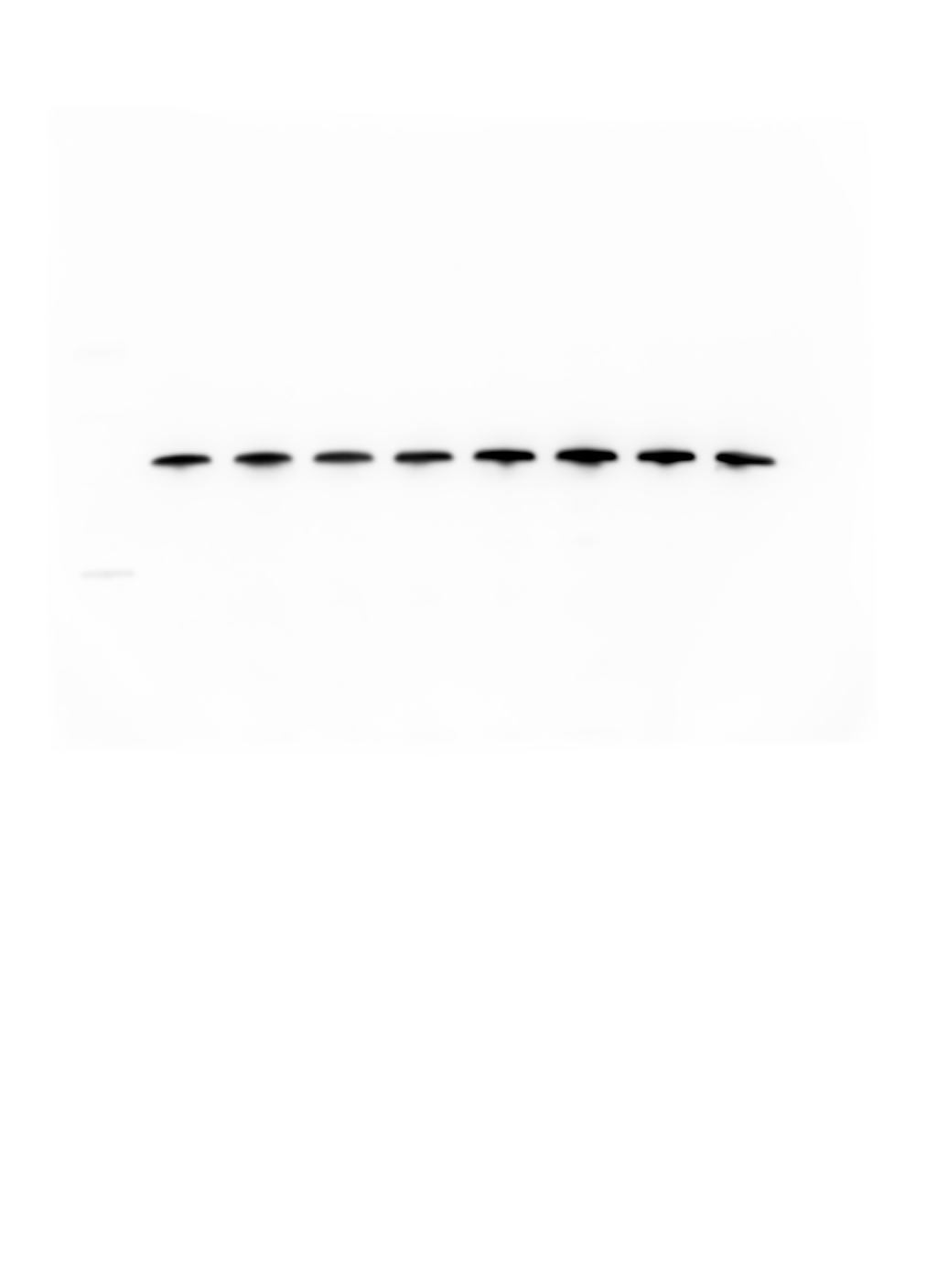

Supplement: Supplementary file 1 [file vaccines-11-00959-s001.zip › file S1-western blot/WB_whole blot/GAPDH/GAPDH-1/20210603-GAPDH-1 2021.06.03_14.07.35_Ch.tif]

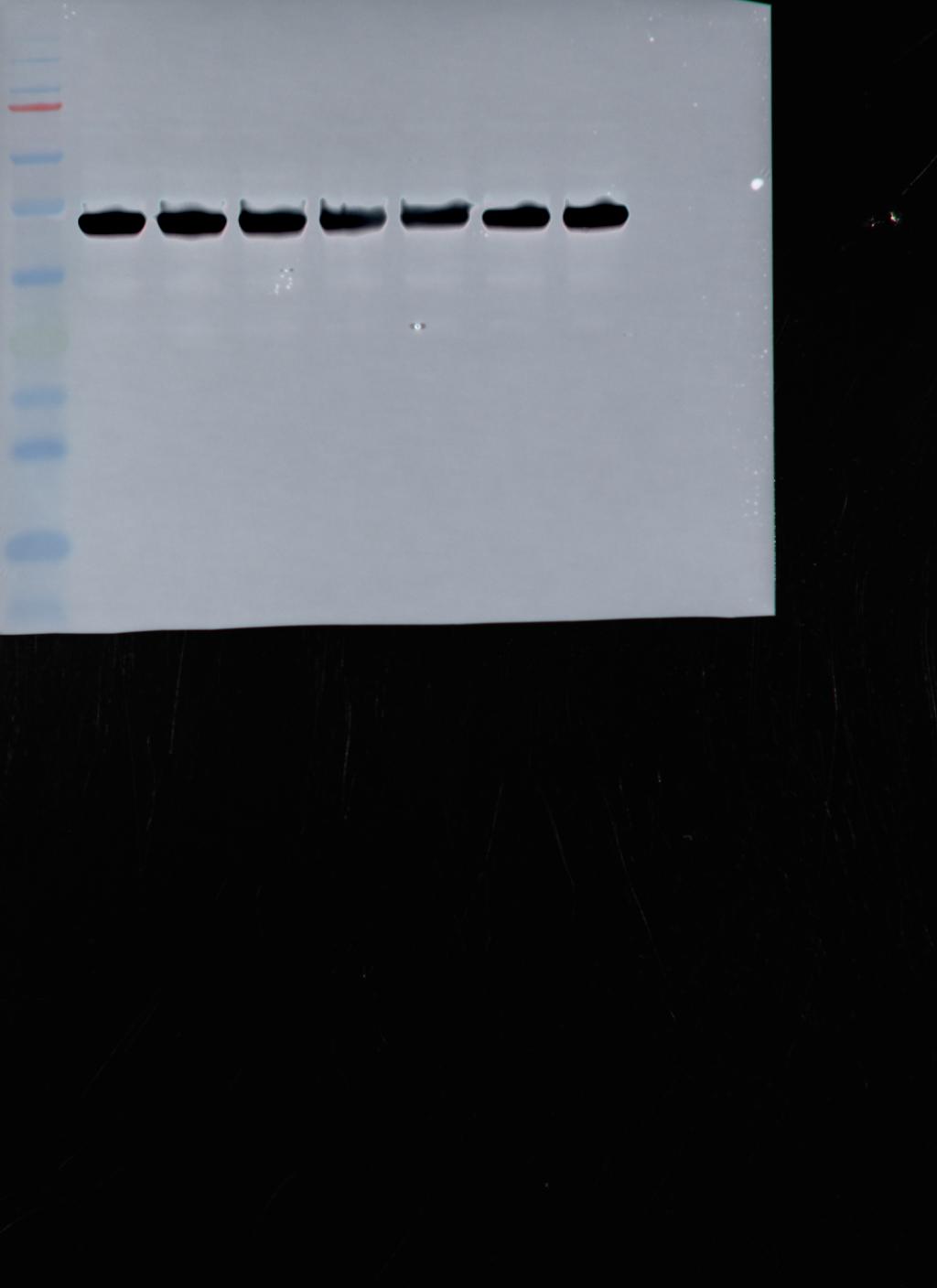

Supplement: Supplementary file 1 [file vaccines-11-00959-s001.zip › file S1-western blot/WB_whole blot/GAPDH/GAPDH-2/20210603-GAPDH-2-2 2021.06.03_14.10.34_Ch+Marker.jpg]

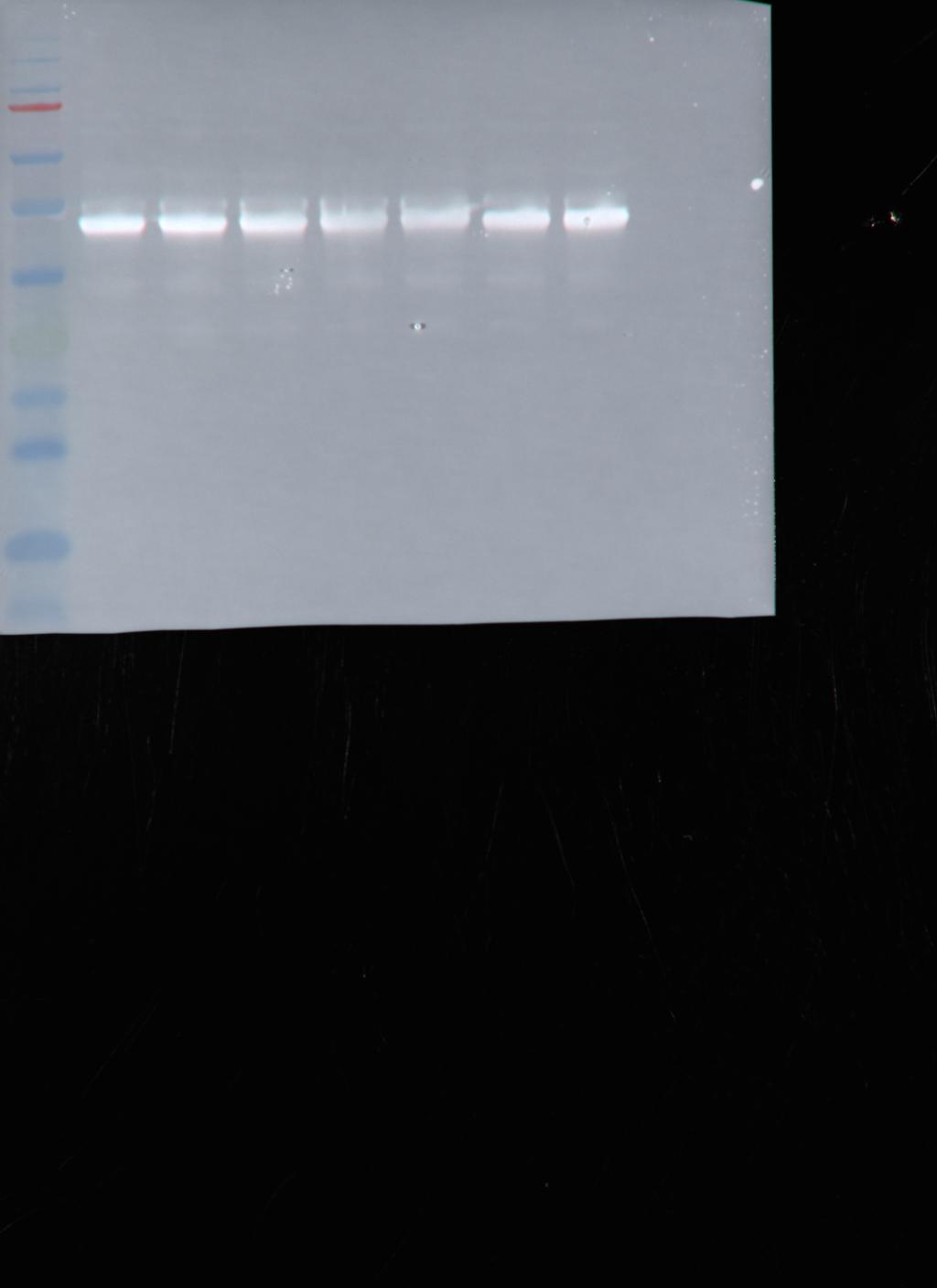

Supplement: Supplementary file 1 [file vaccines-11-00959-s001.zip › file S1-western blot/WB_whole blot/GAPDH/GAPDH-2/20210603-GAPDH-2-2 2021.06.03_14.10.34_Ch-Marker.jpg]

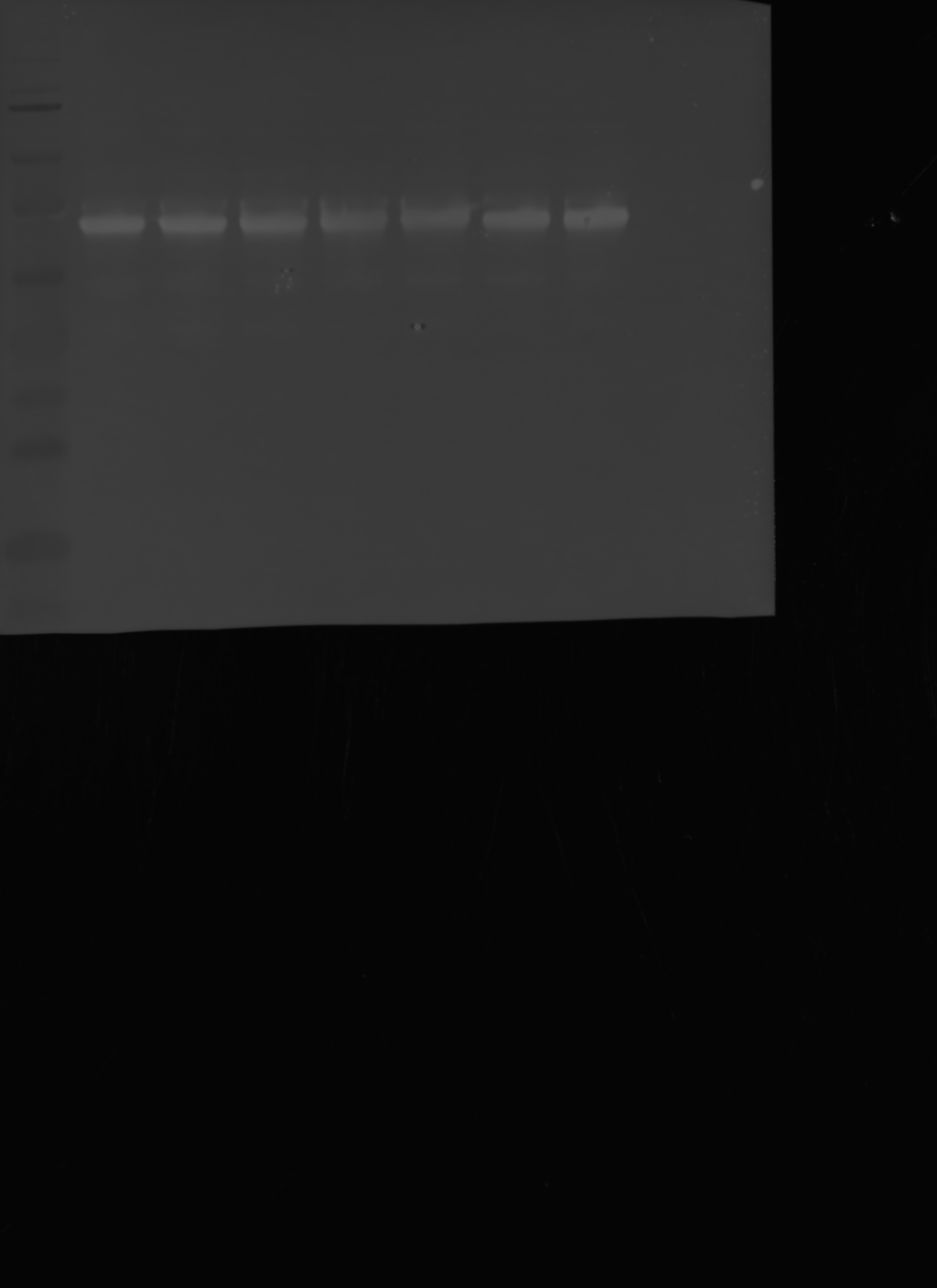

Supplement: Supplementary file 1 [file vaccines-11-00959-s001.zip › file S1-western blot/WB_whole blot/GAPDH/GAPDH-2/20210603-GAPDH-2-2 2021.06.03_14.10.34_Ch-Marker.tif]

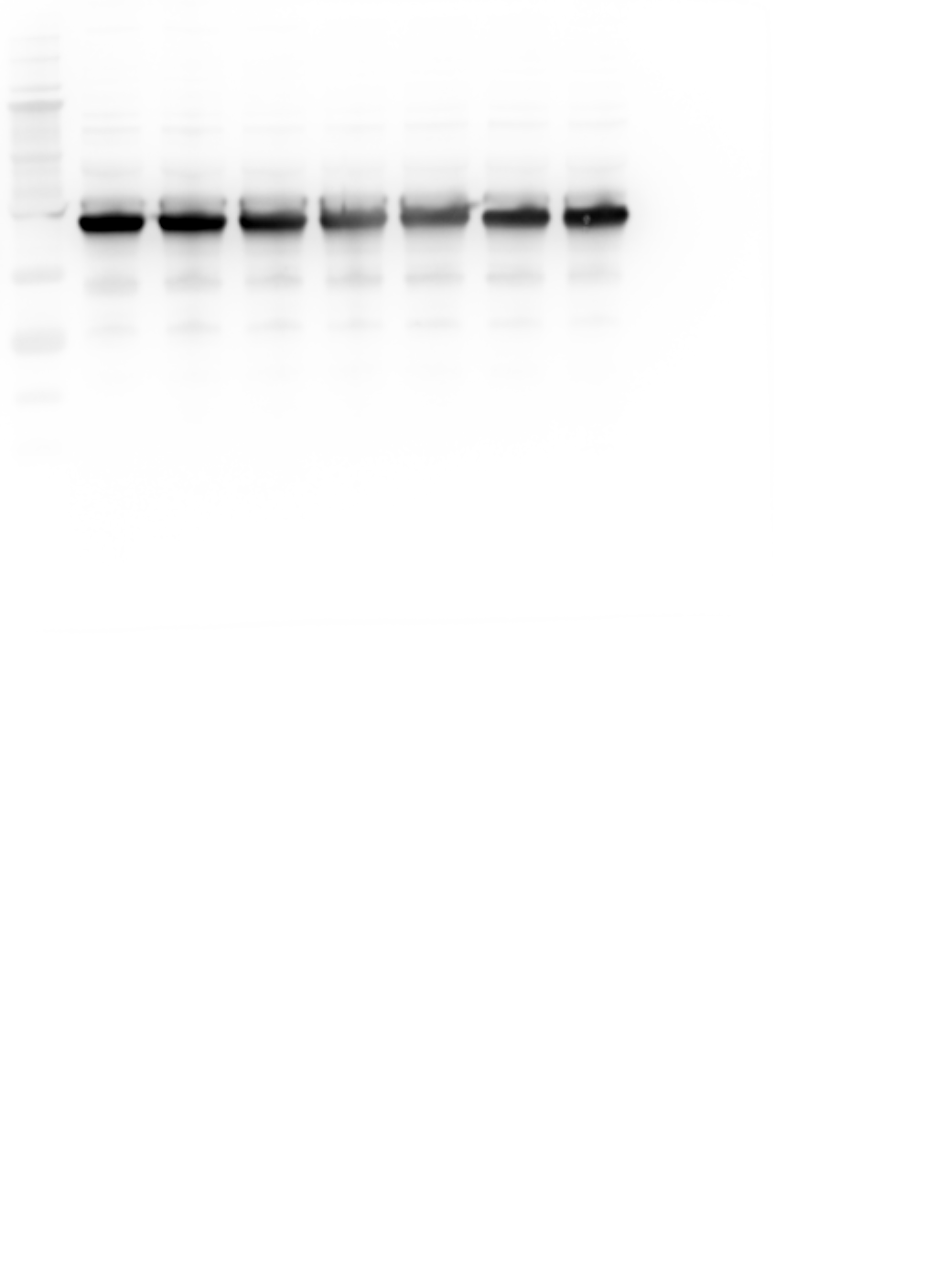

Supplement: Supplementary file 1 [file vaccines-11-00959-s001.zip › file S1-western blot/WB_whole blot/GAPDH/GAPDH-2/20210603-GAPDH-2-2 2021.06.03_14.10.34_Ch.tif]

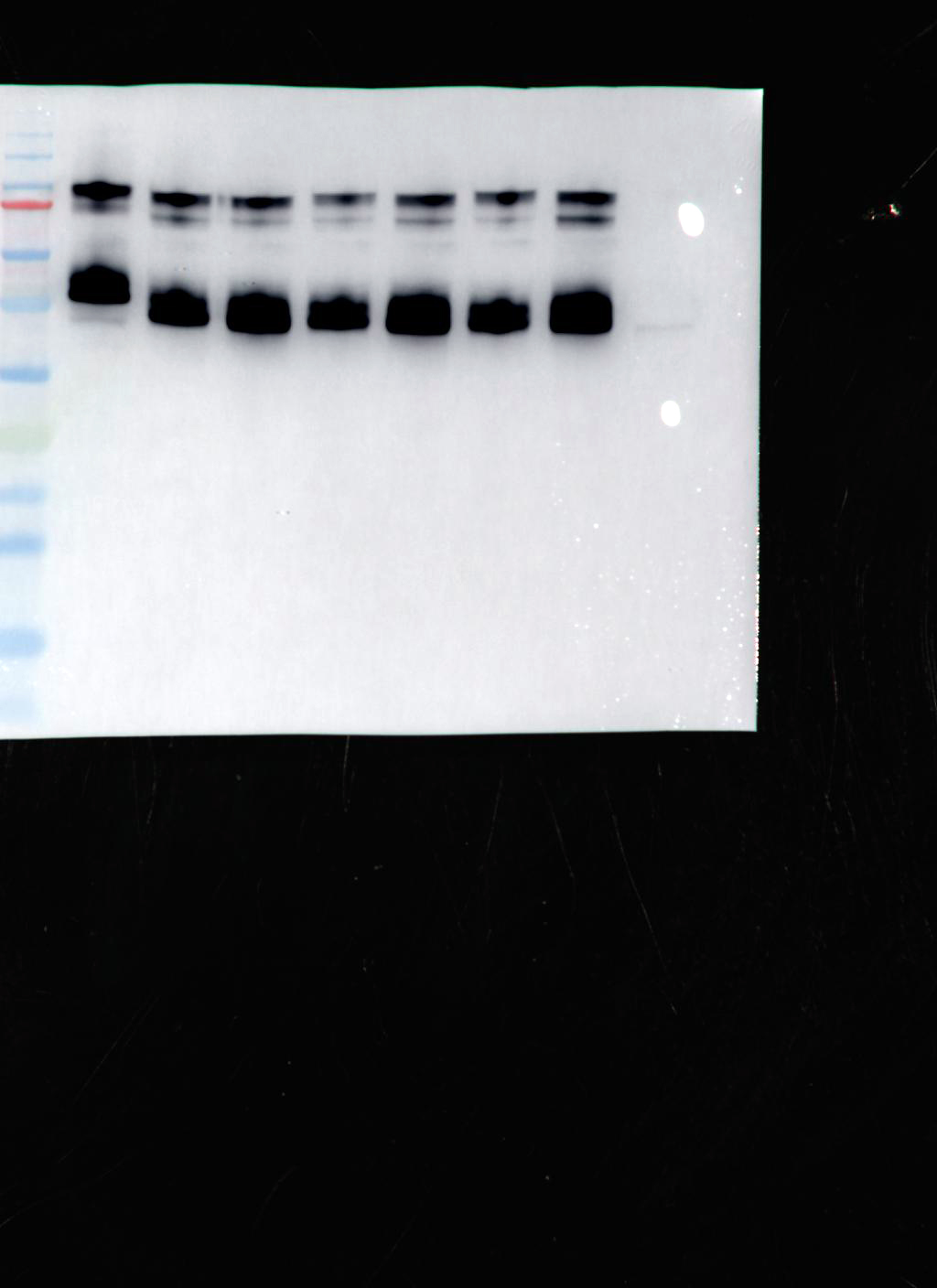

Supplement: Supplementary file 1 [file vaccines-11-00959-s001.zip › file S1-western blot/WB_whole blot/NS1/NS1-1/20210603-NS1-1 2021.06.03_13.46.36_Ch+Marker.jpg]

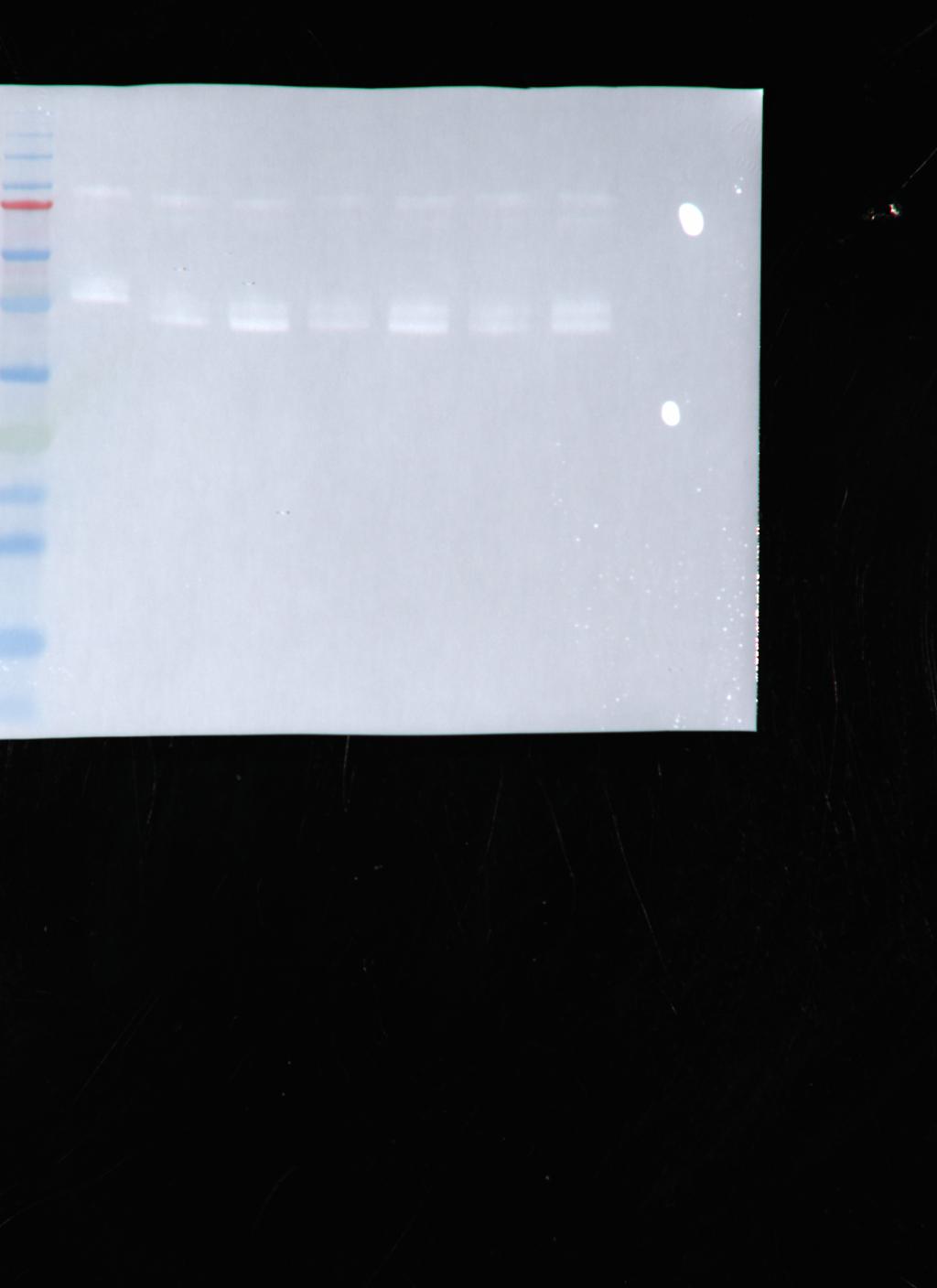

Supplement: Supplementary file 1 [file vaccines-11-00959-s001.zip › file S1-western blot/WB_whole blot/NS1/NS1-1/20210603-NS1-1 2021.06.03_13.46.36_Ch-Marker.jpg]

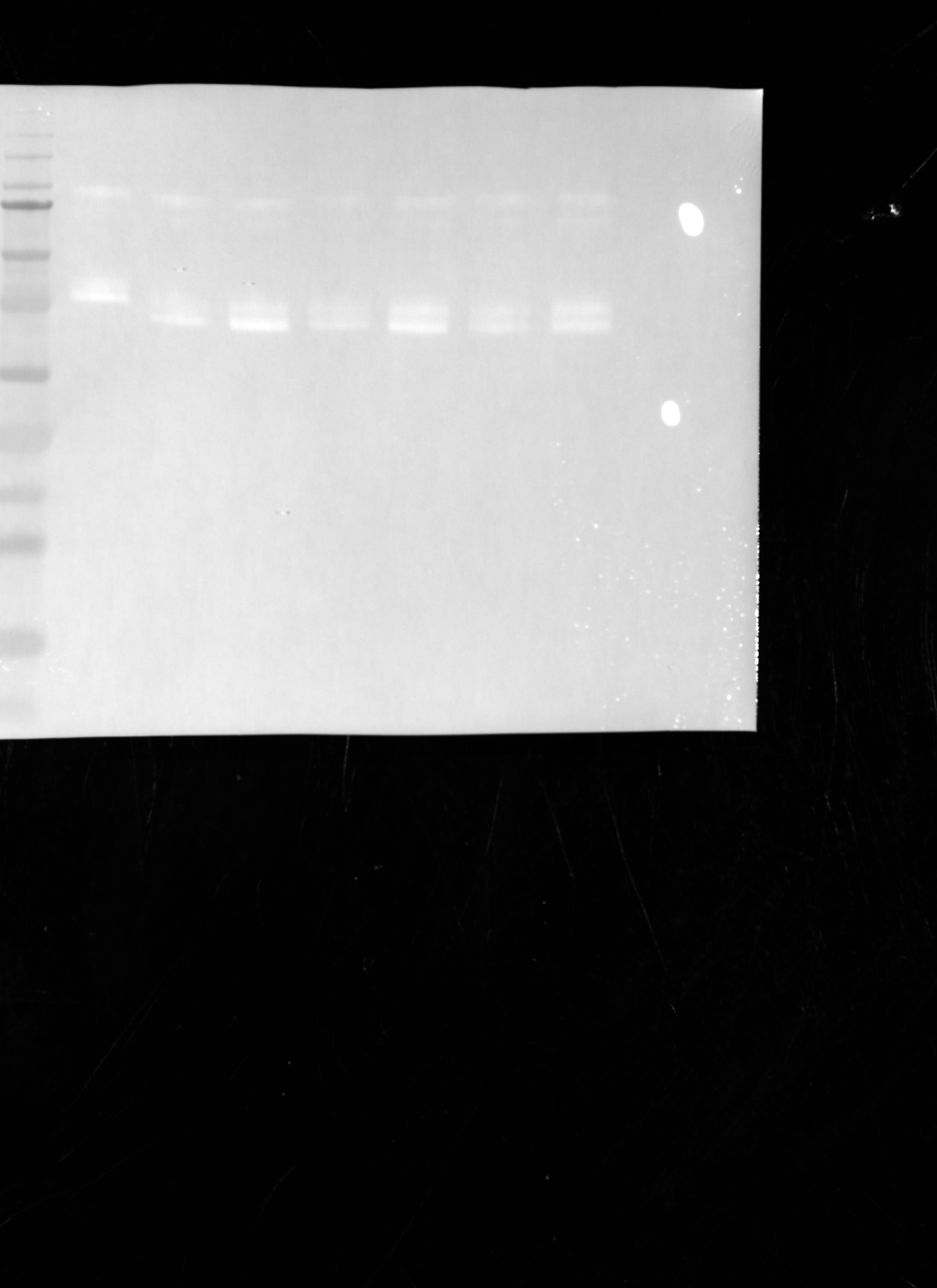

Supplement: Supplementary file 1 [file vaccines-11-00959-s001.zip › file S1-western blot/WB_whole blot/NS1/NS1-1/20210603-NS1-1 2021.06.03_13.46.36_Ch-Marker.tif]

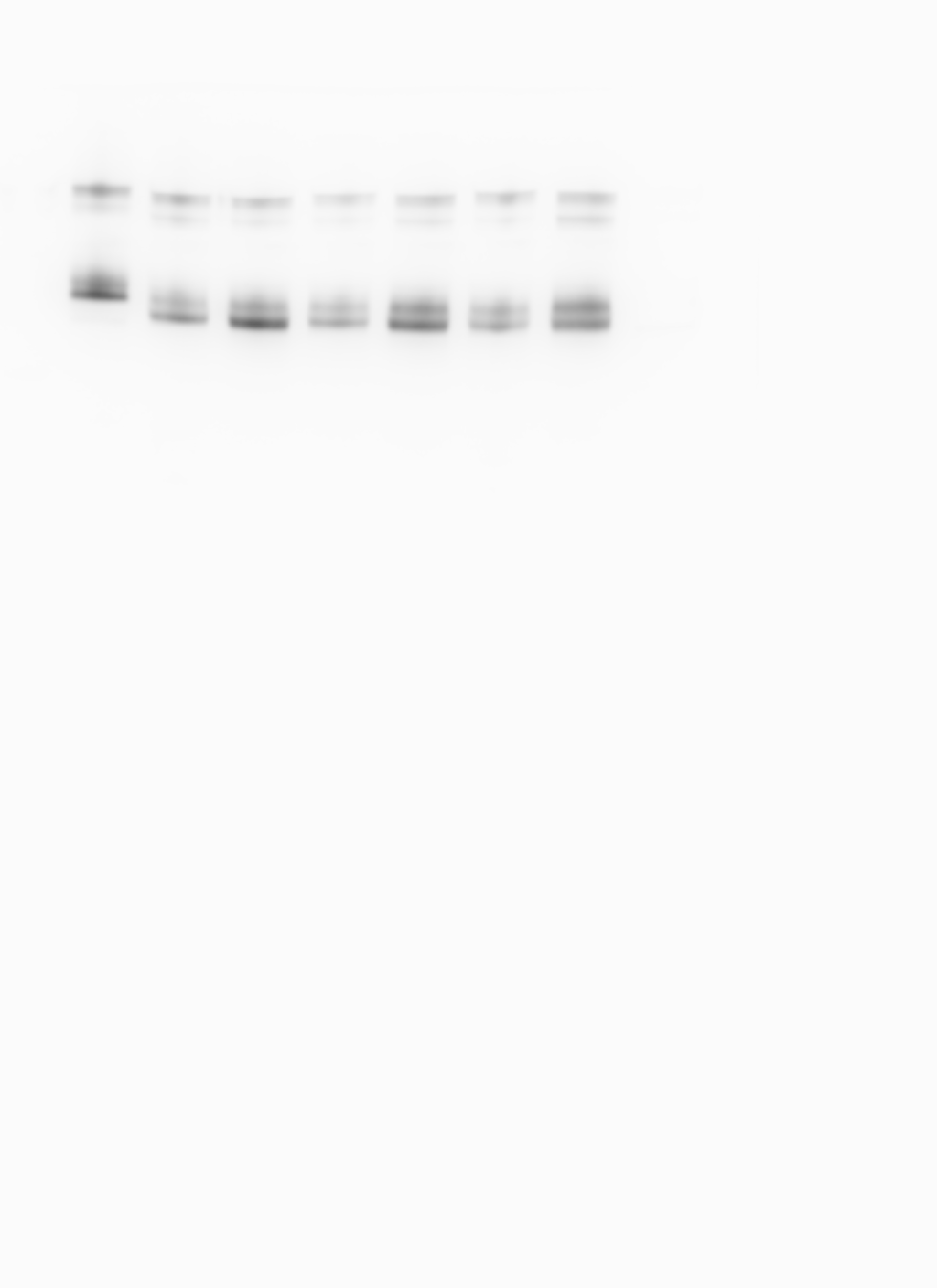

Supplement: Supplementary file 1 [file vaccines-11-00959-s001.zip › file S1-western blot/WB_whole blot/NS1/NS1-1/20210603-NS1-1 2021.06.03_13.46.36_Ch.tif]

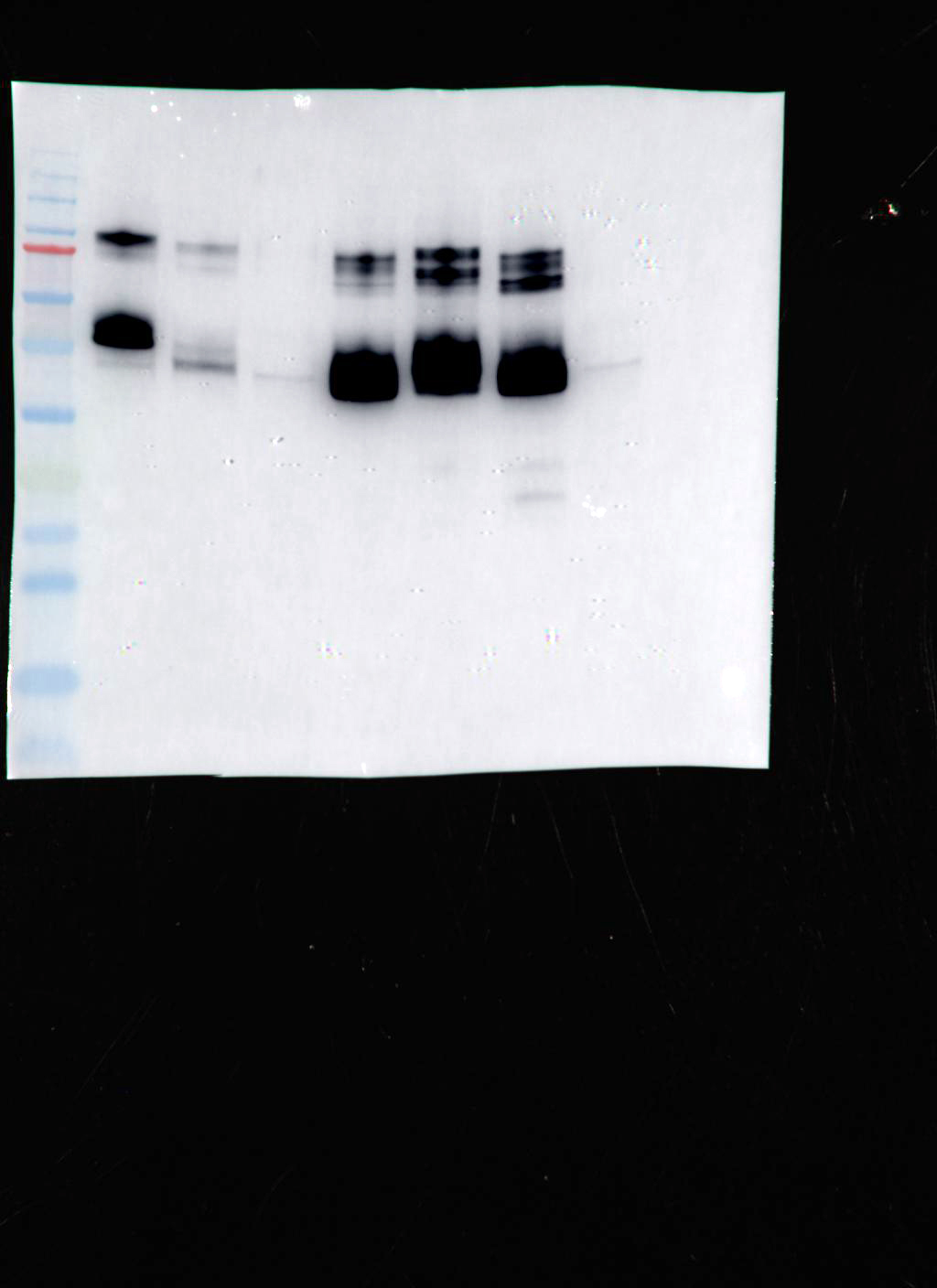

Supplement: Supplementary file 1 [file vaccines-11-00959-s001.zip › file S1-western blot/WB_whole blot/NS1/NS1-2/20210603-NS1-2-2 2021.06.03_13.52.46_Ch+Marker.jpg]

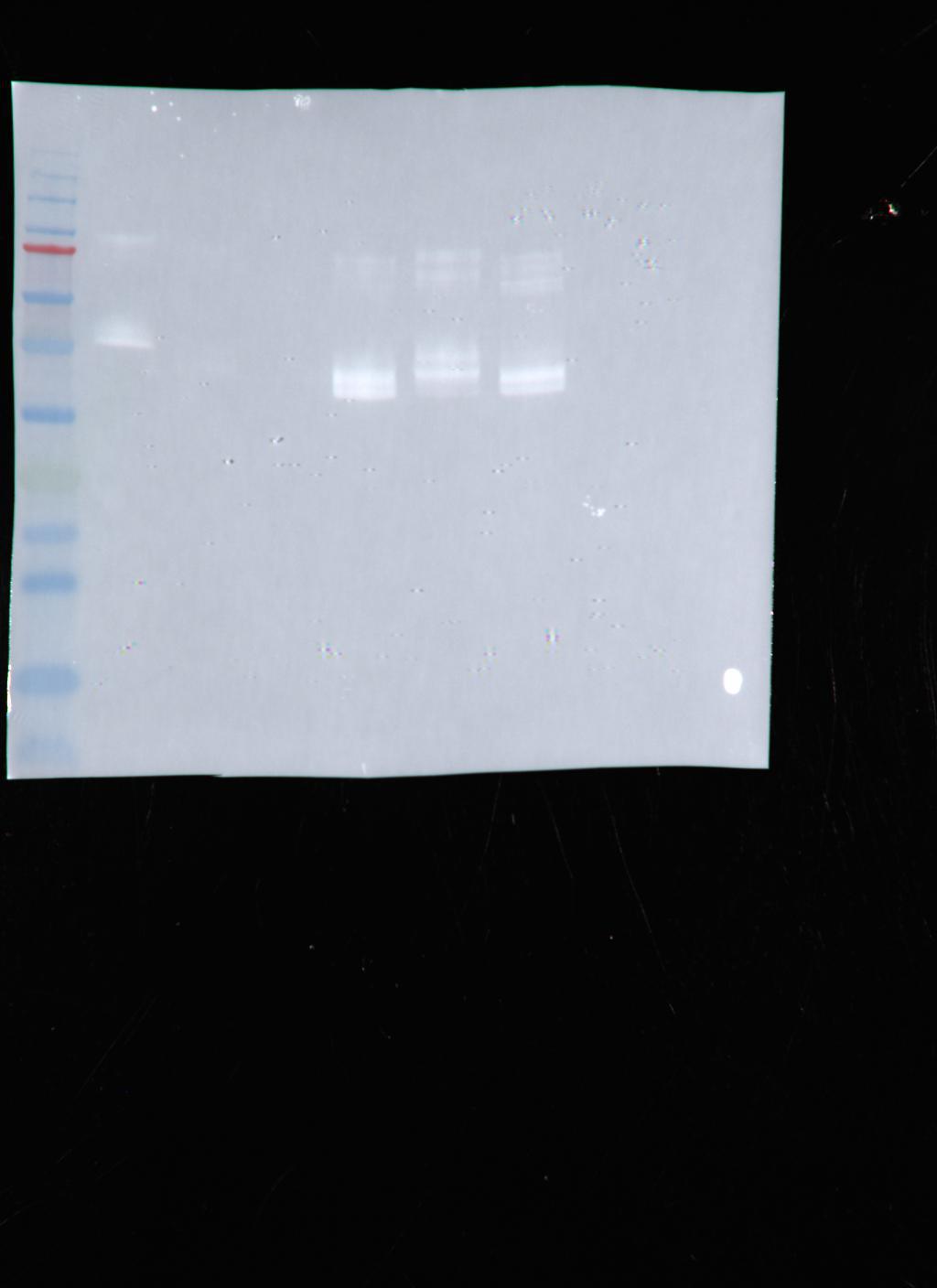

Supplement: Supplementary file 1 [file vaccines-11-00959-s001.zip › file S1-western blot/WB_whole blot/NS1/NS1-2/20210603-NS1-2-2 2021.06.03_13.52.46_Ch-Marker.jpg]

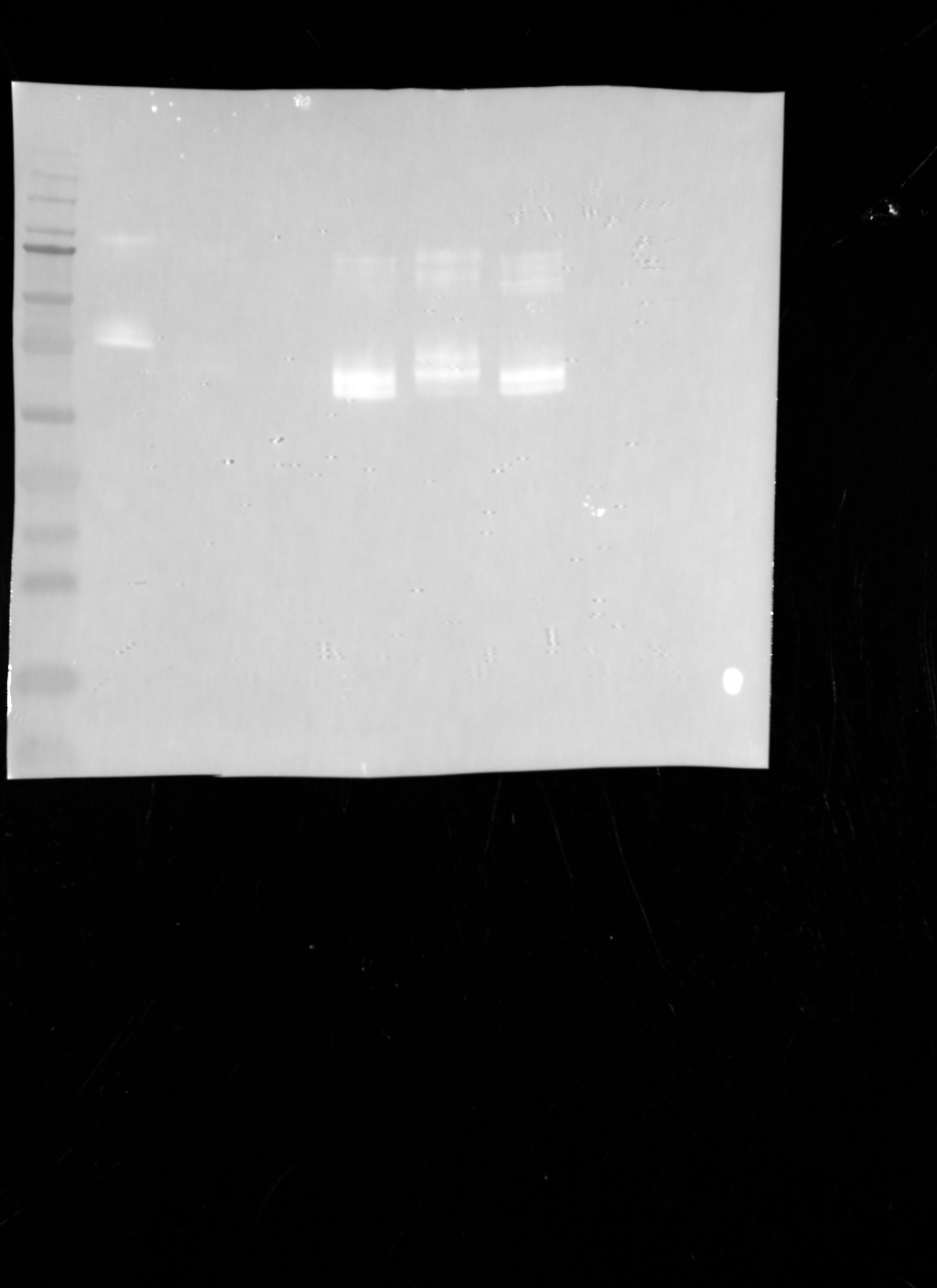

Supplement: Supplementary file 1 [file vaccines-11-00959-s001.zip › file S1-western blot/WB_whole blot/NS1/NS1-2/20210603-NS1-2-2 2021.06.03_13.52.46_Ch-Marker.tif]

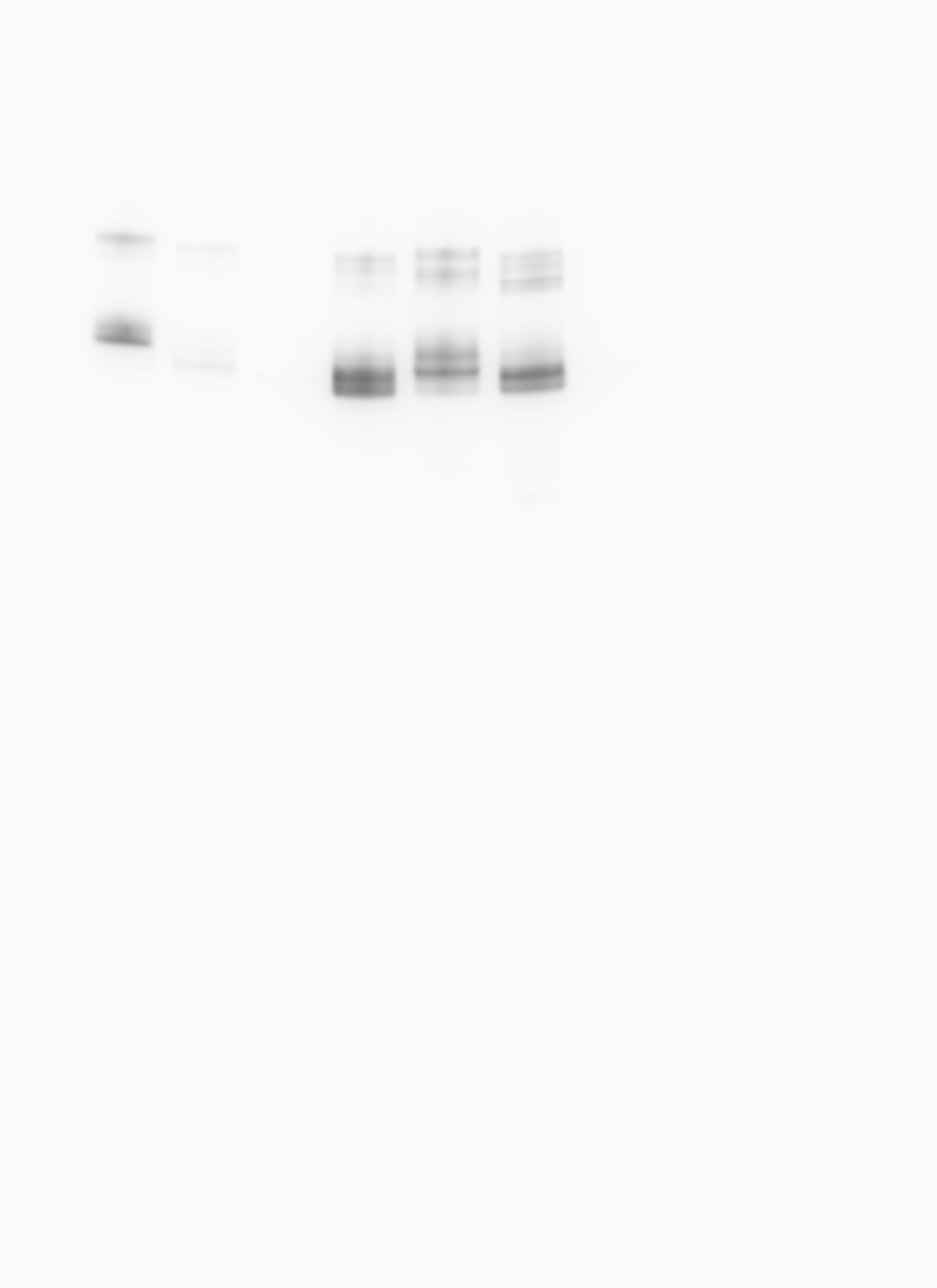

Supplement: Supplementary file 1 [file vaccines-11-00959-s001.zip › file S1-western blot/WB_whole blot/NS1/NS1-2/20210603-NS1-2-2 2021.06.03_13.52.46_Ch.tif]
